# Effect of Stanozolol and/or Cannabis Abuse on Hypertrophic Mechanism and Oxidative Stress of Male Albino Rat Cardiac Tissue in Relation to Exercise: A Sport Abuse Practice

**DOI:** 10.1007/s12012-024-09859-0

**Published:** 2024-05-08

**Authors:** Noha A. Mowaad, Rania Elgohary, Shaimaa ElShebiney

**Affiliations:** Narcotics, Ergogenics and Poisons Department, Medical Research and Clinical Studies Institute, National Research Centre, Dokki, P.O. 12622, Giza, Egypt

**Keywords:** Cardiac hypertrophy, Collagen III (COL3), Doping, Recreational abuse, Sport, Tetrahydrocannabinol (THC), Vascular endothelial growth factor (VEGF-a), Oxidative stress

## Abstract

Adolescents commonly co-abuse many drugs including anabolic androgenic steroids either they are athletes or non-athletes. Stanozolol is the major anabolic used in recent years and was reported grouped with cannabis. The current study aimed at evaluating the biochemical and histopathological changes related to the hypertrophic effects of stanozolol and/or cannabis whether in condition of exercise practice or sedentary conditions. Adult male Wistar albino rats received either stanozolol (5 mg/kg, s.c), cannabis (10 mg/kg, i.p.), and a combination of both once daily for two months. Swimming exercise protocol was applied as a training model. Relative heart weight, oxidative stress biomarkers, cardiac tissue fibrotic markers were evaluated. Left ventricular morphometric analysis and collagen quantification was done. The combined treatment exhibited serious detrimental effects on the heart tissues. It increased heart tissue fibrotic markers (Masson’s trichrome stain (*p* < 0.001), cardiac COL3 (*p* < 0.0001), and VEGF-A (*p* < 0.05)), lowered heart glutathione levels (*p* < 0.05) and dramatically elevated oxidative stress (increased malondialdehyde (*p* < 0.0001) and 8-OHDG (*p* < 0.0001)). Training was not ameliorating for the observed effects. Misuse of cannabis and stanozolol resulted in more hypertrophic consequences of the heart than either drug alone, which were at least largely assigned to oxidative stress, heart tissue fibrotic indicators, histological alterations, and morphometric changes.

## Introduction 

Cannabis has been long associated with athletes and was a component of the “Fuscum Olympionico inscriptum,” or Olympic Victors Dark Ointment, used at the original Olympics as a pain reliever [1]. Cannabis is conceptualized by athletes to be a potent pain-inhibitor [2], where a recent cross-sectional survey study by Zeiger et al. [3] reported that more than 60% of athletes included in the study sample used cannabis at least once in their life and more than 20% continued its use both for pain and recreational reasons. Previous systematic review among adolescent athletes reported the use of recreational cannabis by around 20% of the study participants [4]. Additionally, meta-analysis of 11 studies representing over 46,000 athletes of varying age and ability suggested that around 23% have used some form of cannabis in the past year [5].

The anabolic androgenic steroids (AAS) use among professional athletes and bodybuilders began in the 50s [6]. AAS main medical use is disorders of male hypogonadism and muscle wasting, however, a large percentage of bodybuilders and professional athletes used these agents, at high doses without prescription, to boost muscle growth, energize athletic performance and enhance physical appearance [7]. They usually combine supraphysiological doses of steroids to maximize muscle growth and fat loss. Evidently, AAS users (majorly young adults of age range 18 and 25 accounting for about 50%) engage in polysubstance use [8]. Psychoactive substances such as illegal drugs, alcohol, and addictive prescription drugs are common among these users presumably to reduce the side effects of AAS [9]. In 2021, more than 240 thousand samples were reported into the World Anti-Doping Agency (WADA)’s Anti-Doping Administration and Management System (ADAMS). The report revealed that 4% of the samples were confirmed for cannabinoids (Schedule I) and 40% for AAS use, whereas the major agent (15%) was stanozolol [10], despite being on the prohibited list of WADA. The anxiolytic and pain inhibiting characters of cannabis and the muscle enduring properties of stanozolol are sought by athletes [11].

Stanozolol is the synthetic testosterone 17-alkylated derivative and the most frequently abused AAS. It has a higher anabolic potency and a slower rate of hepatic metabolism than the naturally occurring male hormone. Un-surveilled and non-medical use of AAS entails significant health risks for vital organs including liver and heart toxicities [12]. On the other hand, cannabis contains more than sixty active phytocannabinoids, most importantly tetrahydrocannabinol (THC) and cannabidiol (CBD). THC is a partial agonist at the central CB1 and peripheral CB2 receptors and is considered to be the primary responsible for cannabis-associated psychological effects. Cannabis-associated peripheral risks are underestimated specially by abusers [13]. Moreover, exercise can pose interactions with the endocannabinoid system and modulate the effects of cannabis. Indeed; exercise is known to benefit nearly all physiological systems, including the cardiovascular system. The benefits of exercise depend on the type, frequency, and intensity of the exercise [14]. Athletes are more likely to experience the negative effects of exercise-induced free radical flow, particularly when their workouts are long and intense, however athletes and regular exercisers eventually become adapted to such training programs and become less susceptible to oxidative damage [15] Antioxidant enzyme activity often rises dramatically in response to exercise halting the oxidative damage brought on by exercise-induced lipid peroxidation [16]. The use of muscle boosting AAS along with purposeful or recreational cannabis misuse in the context of sport should be considered for the potential to alter physical performance and the potential for adverse health effects, including serious cardiovascular events, which is the main particular purpose of this study. There are a few literatures that study the combined effect of both cannabis and stanozolol in athletes, so this research was oriented to fill this gap.

## Materials and Methods

### Drugs and Chemicals

Stanozolol vials were purchased (ZPHC. Lic No; 1839-2010/ILS, USA). Cannabis resin was obtained from the department of Azbakeyya Criminalistics, Ministry of Justice case no. 809/2019 (Cairo, Egypt). The resin was chloroform extracted, dried, and pulverized. 10 mg/kg of the dried powdered resin was dispersed in saline and injected intraperitoneally (i.p.) [17].

### Experimental Design

Forty-eight male adult Wistar rats weighing about (200–250 mg, 7 months old) were obtained from the National Research Centre breeding colony, Cairo, Egypt. The animals were housed in polypropylene cages (6/cage) and kept in a 12-h dark/light cycle at a temperature of 20–23 °C. Animals were fed with commercial high protein rat diet pellets ad libitum and were given tap water. They were left to acclimatize in the experimental room for 1 week before carrying out any procedure. This study was ethically approved by the Institutional Animal Care and Use Committee (IACUC), NRC (CU-II-F-10-18) and followed the National Institutes of Health Guide Recommendations Care and Use of Laboratory Animals (Publication No. 85-23, revised 1985).

Rats were assigned to 8 different groups (*n* = 6 for each group). Four groups were trained on swimming protocol, while the other four groups were left sedentary. Group 1 was treated with saline (i.p.), Group 2 was trained and treated with stanozolol (5 mg/kg, s.c) [18], one hour before each exercise session. Group 3 was trained and treated with cannabis resin extract (10 mg/kg, i.p.), one hour before each exercise session, and Group 4 was trained and treated with stanozolol (5 mg/kg, s.c) and cannabis (10 mg/kg, i.p.), one hour before each exercise session. Sedentary groups [5–8] received the same corresponding treatments without being trained.

### Swimming Protocol

Swimming was applied as an aerobic exercise model in rats and was adapted from the protocol applied by Barbosa dos Santos et al. [19]. The rats were trained to swim in a cylindrical tank with dimensions of 85 × 50 × 60 cm filled with water at 30–31 °C. Exercise sessions began between 10:00 and 11:00 in the morning. Prior to the start of experiment, rats were subjected to a week of adaptive training where the animals were kept in shallow water to get familiar with water immersion and eliminate stress conditions. The time was increased by 5 min each day, starting at 10 min and increasing the water depth by 5 cm each day. Continuous exercise training was conducted after the adjustment period for 20 min/day, 5 days/week, for 8 consecutive weeks.

### Sample Collection and Preparation

At the end of the experimental period, animals were weighed, euthanized under light anesthesia, and heart tissue was excised, trimmed of adhering structures, washed by saline, blotted dry on filter paper, and weighed immediately.

The entire heart was cut into two vertical sections, the first section was immediately frozen in liquid nitrogen and subsequently stored at − 80 °C, while the other section was stored in 10% formalin for 72 h then dehydrated and embedded in paraffin blocks for histological examination. Frozen heart samples were homogenized in ice-cold phosphate buffer saline (pH7.4), and then centrifuged at 4000 rpm for 15 min at 4 °C for further biochemical analysis. The relative heart weight (RHW) index was calculated as heart weight/body weight × 100 (%).

### Biochemical Analysis

Spectrophotometric methods using standard laboratory reagents were used to determine the cardiac tissue levels of MDA [20], GSH [21], and nitric oxide (NO) [22].

Standard ELISA kits were used for the in vitro quantitative determination of 8-hydroxydeoxyguanosine (8-OHdG) (Cat#E-EL-0028) Collagen type III (COL3) (Cat#E-EL-R0235) and vascular endothelial growth factor (VEGF-A) (Cat#E-EL-R 2603), and procured from Elabscience, Wuhan, China.

### Histological Examination

Heart tissue samples processing and staining was done according to Culling [23]. Tissue sections of 4 μm thick were cut and mounted on glass slides to demonstrate full cardiac wall thickness. Other sections were stained with Hematoxylin and Eosin and Masson’s trichrome stain to demonstrate collagen deposition. Six representative non-overlapping fields were randomly selected per tissue section of each sample for the determination of left ventricular wall thickness and the area percentage of collagen fiber deposition. Data were obtained using Full HD microscopic imaging system (Leica Microsystems GmbH, Germany) operated by Leica Application software for tissue sections analysis at magnification of 100× and ×400×.

### Statistical Analysis

Data were analyzed by a two-way ANOVA test for the two main effects (exercise training and drug treatment) and for the interaction between drugs. To determine differences across groups, analysis of variance (ANOVA) and Tukey’s test were used to calculate statistical differences. Analysis was performed using Graphpad Prism software (Graphpad Software Inc., USA). The findings are presented as means with standard error, and differences were deemed significant at *p* < 0.05.

## Results

### Effect of Stanozolol and Cannabis Misuse on Body Weight and Relative Heart Weight (RHW)

Table 1 presents the body weight of different experimental groups at week 0 and week 8 and the relative heart weight after the end of the experimental period. The two-way ANOVA revealed no significant interaction or effect for the body weight gain during the experimental period (*p* = 0.32). On the other hand, there was a significant interaction between exercise and RHW *F*(1, 40) = 11.78, *p* = 0.0014 and between drug treatment and RHW *F*(3, 40) = 17.44, *p* < 0.0001. In sedentary animals, administration of cannabis or both substances in sedentary animals resulted in an increased RHW compared to sedentary control rats (+ 32, and 45%, *p* < 0.05). Likewise, multiple comparison test of RHW revealed a significant difference (*p* < 0.05) of RHW after the use of stanozolol (+ 36%) or both substances (+ 48%) in trained animals (Table 1).

**Table 1:** Effect of stanozolol and cannabis misuse on body weight gain and relative heart weight in sedentary and trained rats

	Control	Stanozolol	Cannabis	Combined
Weight gain percentage (%)				
Sedentary	32.0 ± 6.60	24.0 ± 8.40	14.0 ± 8.20	14.0 ± 2.70
Trained	18.0 ± 3.90	21.0 ± 6.50	17.0 ± 7.10	12.0 ± 2.80
Weight at week 0 (g)				
Sedentary	274.83 ± 12.41	267.66 ± $11.78$	269.33 ± $12.54$	264.5 ± $7.42$
Trained	261 ± 5.9	253.33 ± $6.09$	263.66 ± $12.64$	253 ± $9.36$
Weight at week 8 (g)				
Sedentary	346 ± $12.1$	332.33 ± 15.9	308.16 ± 16.9	300.83 ± 3.1
Trained	307 ± 5.06	307.6 ± 14.03	307.2 ± 11.6	283 ± 9.36
Relative heart weight (mg/g tissue)				
Sedentary	2.23 ± $0.08$	2.78 ± $0.13$	2.92 ± $0.16$^a^	3.27 ± 0.11^a^
Trained	2.58 ± $0.10$	3.41 ± 0.20^a^	2.83 ± $0.15$	3.70 ± $0.21$^a^
Heart weight (g)				
Sedentary	0.77 ± 0.01	0.92 ± $0.02$^a^	0.90 ± $0.01$^a^	0.98 ± $0.02$^a^
Trained	0.79 ± 0.02	1.04 ± $0.04$^a^	0.87 ± $0.02$	1.04 ± $0.03$^a^

Animals were administered stanozolol 5 mg/kg, s.c., or cannabis resin extract 10 mg/kg, i.p., or both substances for a period of 8 weeks. A set of groups was kept sedentary and the other set was trained by swimming protocol. The results are expressed as mean ± SEM; Significance was tested at *p* < 0.05 using two-way ANOVA followed by Tukey’s post hoc test for comparison (*n* = 6/group).

^a^Compared to the corresponding set control

### Effect of Stanozolol and Cannabis Misuse on Cardiac Tissue Fibrotic Biomarkers (Collagen Type III, VEGF-A)

**Fig. 1:**
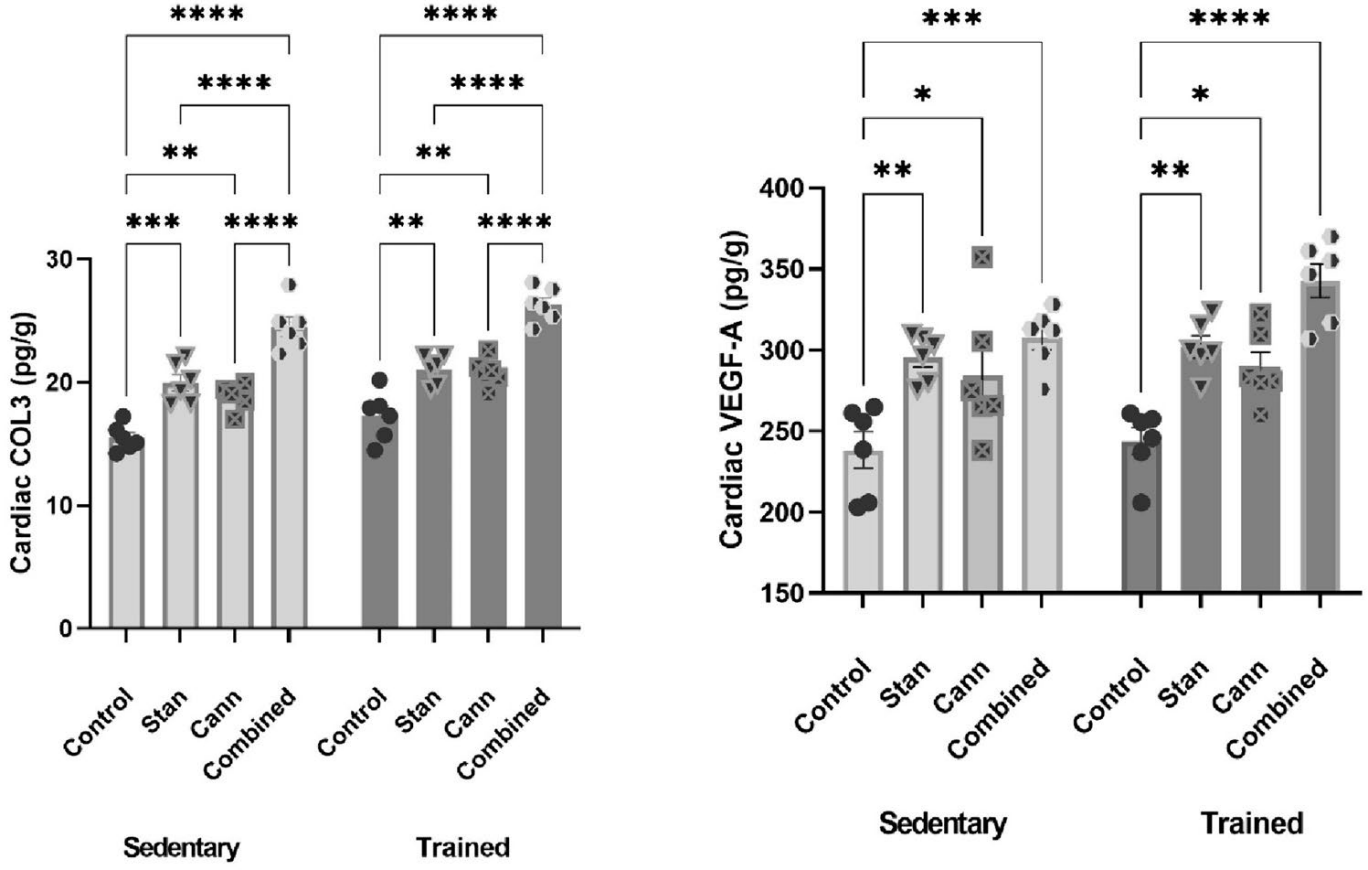
Effects of stanozolol, cannabis, or combined administration on cardiac collagen type III (COL3) and vascular endothelial growth factor (VEGF-A) in sedentary and trained rats. Training was of insignificant effect, while different treatments increased the accumulation of fibrotic biomarkers significantly in cardiac tissue of sedentary and trained rats. Rats were administered stanozolol 5 mg/kg, s.c. (Stan), or cannabis resin extract 10 mg/kg, i.p. (Cann), or both substances (Combined) for a period of 8 weeks. A set of groups was kept sedentary and the other set was trained by swimming protocol. The results are expressed as mean ± SEM; Significance was tested at *p* < 0.05 using two-way ANOVA followed by Tukey’s post hoc test for comparison (*n* = 6/group). *, **, ***, **** compared to the corresponding set control at *p* < 0.05, 0.01, 0.001, 0.0001, respectively

Two-Way ANOVA of COL3 data showed no interaction between training and treatments *F*(3, 40) = 0.08, *p* = 0.97, while training affected the accumulation of COL3 in cardiac tissue *F*(1, 40) = 13.36, *p* = 0.0007 and there was a significant interaction between treatments *F*(3, 40) = 77.80, *p* < 0.0001 (Fig. 1). Multiple comparisons of data after different treatments revealed that the COL3 content of sedentary rats treated with stanozolol, cannabis, or combined treatments was significantly affected (Mean difference between sedentary control and sedentary stanozolol = − 4.95, 95% CI − 7.846 to − 2.06, *p* < 0.0001, mean difference between sedentary control and sedentary cannabis = − 3.29, 95% CI − 6.19 to − 0.41,* p* < 0.0001, mean difference between sedentary control and sedentary combined = − 9.30, 95% CI − 12.19 to − 6.41, *p* < 0.0001) (Fig. 1).

Different treatments exhibited a significant increase in COL3 in heart tissue of trained rats (Mean difference between trained control and trained stanozolol = − 5.31, 95% CI − 8.21 to − 2.42, *p* < 0.0001, mean difference between trained control and trained cannabis = − 3.39, 95% CI − 6.27 to − 0.49, *p* < 0.05, mean difference between trained control and trained combined = − 9.85, 95% CI − 12.74 to − 6.96, *p* < 0.0001). On the other hand, training was of no significant effect when comparing cardiac COL3 in trained control rats to sedentary rats (mean difference = 1.41, 95% CI − 1.48 to 4.29, *p* > 0.05) (Fig. 1).

The two-way ANOVA of VEGF-A data revealed that factorial interaction accounts for 2.7% of the total variance *F*(3, 40) = 1.08, *p* = 0.37, while treatment effect accounts for 61% of the total variance *F*(3, 40) = 24.22, *p* < 0.0001. Training was not quite significant, *F*(1, 40) = 3.34, *p* = 0.07. Mean difference between sedentary control and trained control rats = − 5.478, 95% CI − 51.11 to 40.16, *p* > 0.05 (Fig. 1). Multiple comparisons of sedentary rats after receiving different treatments revealed significant difference (Mean difference between sedentary control and sedentary stanozolol = − 57.20, 95% CI − 102.8 to − 11.56, *p* = 0.006, Mean difference between sedentary control and sedentary cannabis = − 46.21, 95% CI − 91.84 to − 0.57, *p* = 0.04, Mean difference between sedentary control and sedentary combined = − 69.23, 95% CI − 114.9 to − 23.59, *p* = 0.0005) (Fig. 1). Likewise, significant effect of different treatments was observed in trained rats (Mean difference between trained control and trained stanozolol = − 58.20, 95% CI − 103.8 to − 12.57, *p* = 0.005, Mean difference between trained control and trained cannabis = − 45.69, 95% CI − 91.33 to − 0.06, *p* = 0.05, Mean difference between trained control and trained combined = − 99.02, 95% CI − 144.7 to − 53.38, *p* < 0.0001) (Fig. 1).

### Effect of Stanozolol and Cannabis Misuse on Cardiac Tissue Histological Features and Collagen Fibers

**Fig. 2:**
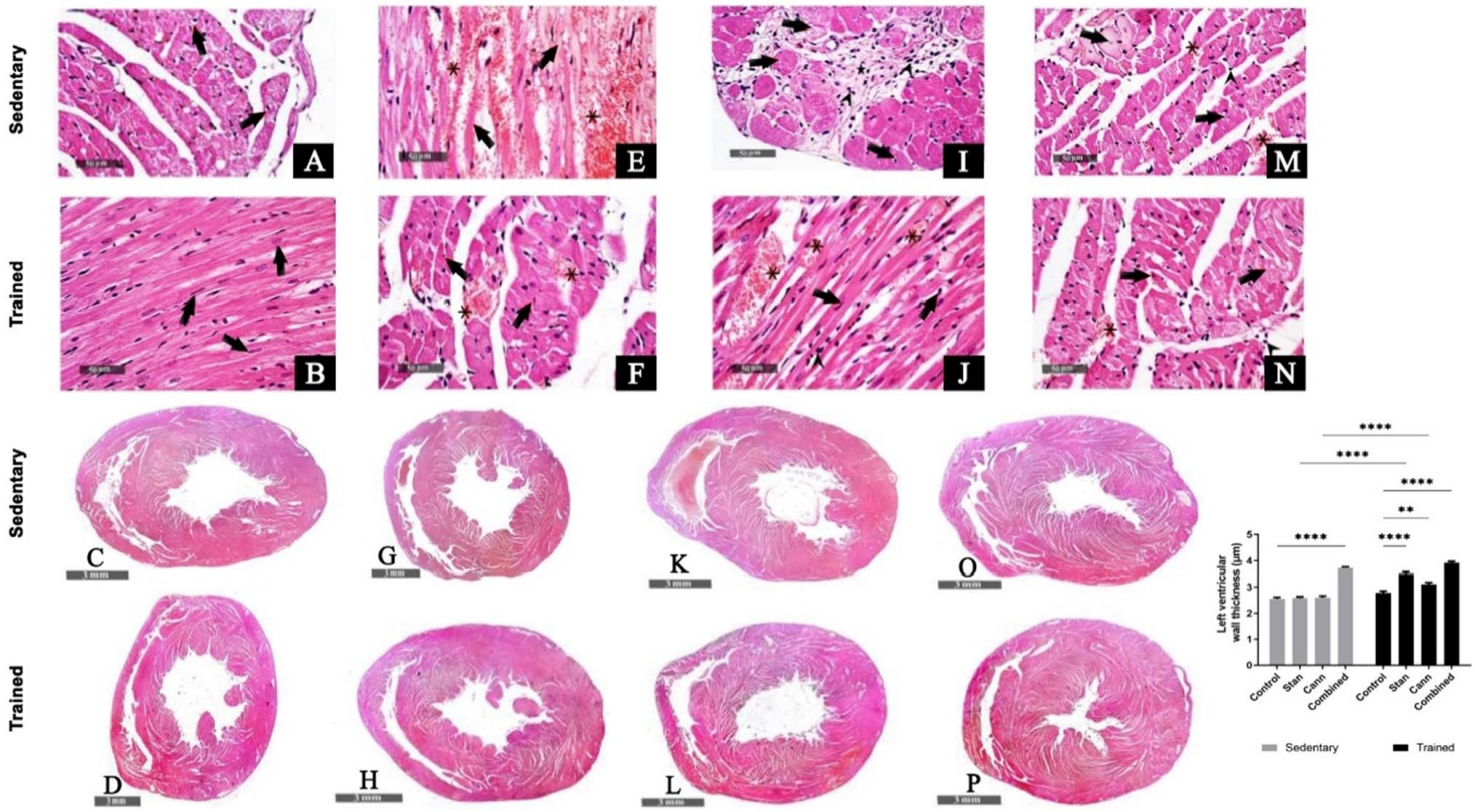
Examination of H&E-stained heart sections under light microscope of sedentary and trained rats showing degenerative changes (arrow) and focal hemorrhage (star). Left ventricular wall thickness. Stanozolol administration (**E**–**H**), cannabis administration (**I**–**L**), and both substances administration (**M**–**P**) presented focal subendocardial degeneration and necrotic cardiomyocytes with nuclear pyknosis (arrow), moderate increased intermuscular spaces with congested vasculatures (star), and increased fibroblastic activity that were more prominent in the combined group. Left ventricular wall thickness data were obtained using Full HD microscopic imaging system (Leica Microsystems GmbH, Germany) operated by Leica Application software for tissue sections analysis and representative samples were selected for imaging. Data in graph are presented as mean ± SEM and tested for significance at *p* < 0.05 using two-way ANOVA followed by Tukey's multicomparison test (*n* = 6)

Swimming training had no effect on the morphological features of the heart tissue. The light microscopic examination of the cardiac tissue of sedentary control rats showed intact, well organized morphological features of cardiac wall layers and minimal sporadic records of degenerative changes (Fig. 2A). Training did not change the histological features of the cardiac wall layers where the microscopic examination of heart tissue sections of trained control animals displayed intact, well organized branched cardiomyocytes and subcellular details with intact vasculatures without abnormal cellular infiltrates (arrow) (Fig. 2B). The left ventricular wall thickness was not changed after training of control rats compared to sedentary control rats (Fig. 2C and D).

Demonstrated moderate hypertrophy of cardiac wall thickness with focal sever records of necrotic cardiomyocytes (arrow) accompanied with marked intermuscular hemorrhage (star) in stanozolol sedentary rats (Fig. 2E). Stanozolol trained rats showed focal subendocardial figures of degenerated and necrotic cardiomyocytes with nuclear pyknosis (arrow) and moderate increased intermuscular spaces with congested vasculatures (star), with mild increased fibroblastic activity after stanozolol administration to sedentary and trained rats (Fig. 2F). This was reflected as increased left ventricular wall thickness observed after stanozolol administration to sedentary and trained rats in comparison to control rats (Fig. 2G and H).

Cannabis administration showed marked hypertrophy of left ventricular wall thickness with focal subendocardial fibrotic lesions (star), mononuclear inflammatory cells infiltrates (arrow head) associated with many figures of degenerated cardiomyocytes with nuclear pyknosis (arrow) and moderate intermuscular hemorrhage (star) (Fig. 2I–L).

Both substances administration to sedentary or trained rats resulted great hypertrophy of left ventricular wall thickness with mild fibroblastic activity, persistent moderate records of degenerative and necrotic changes in cardiomyocytes (arrow) with moderate congested intermuscular BVs (star). Great thickness of the left ventricular wall associated with persistent degenerative and necrotic fibroblastic activity and prominent congested intermuscular blood vessels (Fig. 2M–P).

**Fig. 3:**
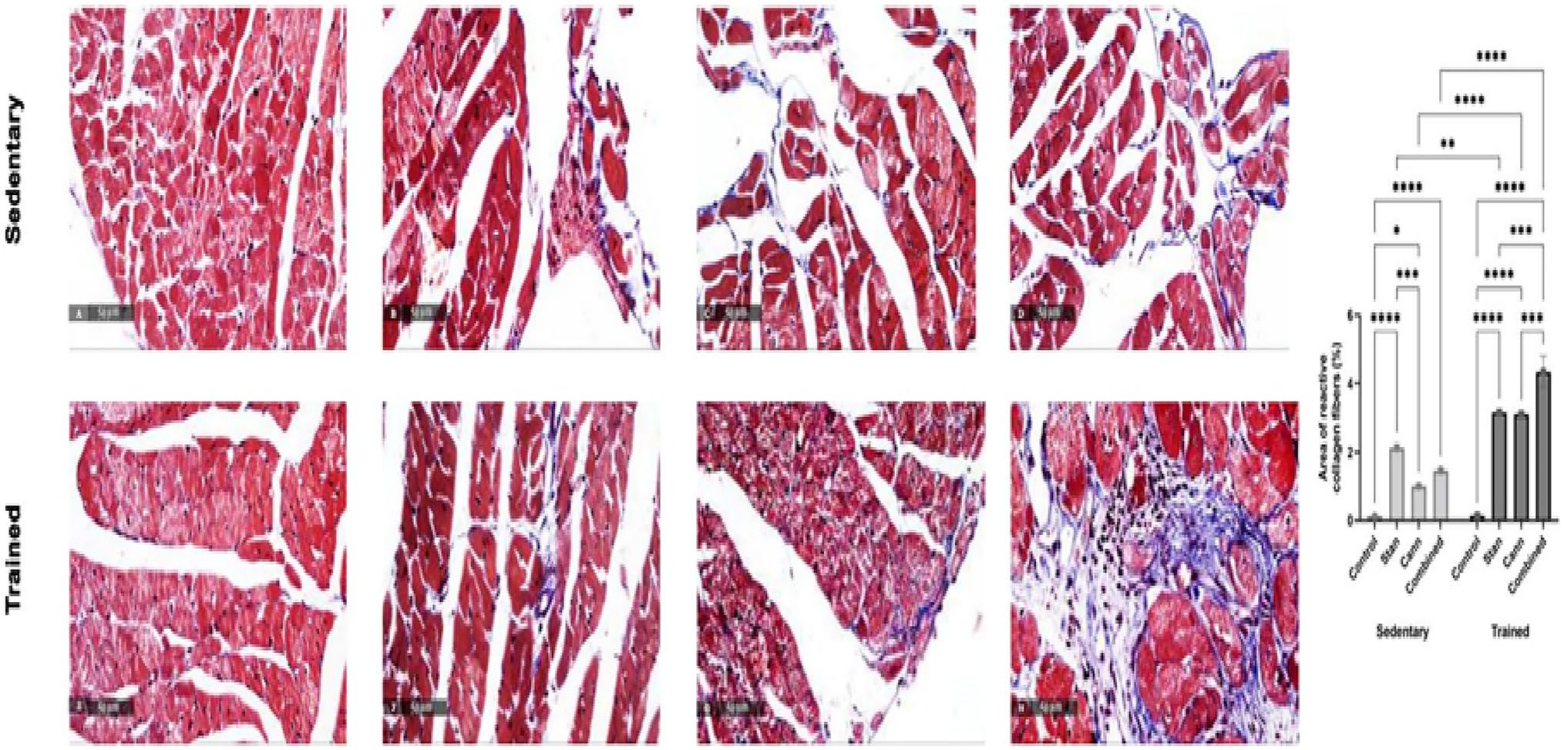
Masson’s Trichrome stain of deposited collagen fibers in interstitial connective tissue. Significant accumulation of collagen fibers (blue stain) was observed after stanozolol (**B**, **F**), cannabis (**C**, **G**), or both substances (**D**, **H**) administration to sedentary (upper row) and trained (lower row) rats. Training resulted in more significant deposition in treated rats. Six non-overlapping sections were examined and collagen deposited area percentage was determined using Full HD microscopic imaging system (Leica Microsystems GmbH, Germany) operated by Leica Application software for tissue sections analysis at magnification of ×100 and ×400. Data in graph are presented as mean ± SEM and tested for significance at *p* < 0.05 using two-way ANOVA followed by Tukey's multicomparison test (*n* = 6)

Accumulated collagen fibers in interstitial connective tissue were observed by histological examination of Masson’s trichrome stained heart tissue sections. Stained area estimation of the deposited collagen showed a significant (*p* < 0.05) increase after stanozolol (2.1 ± 0.1%), cannabis (1.0 ± 0.1%), and combined treatments (2.5 ± 0.1%) compared to control (0.11 ± 0.04%) sedentary rats (Fig. 3A–D). On the other hand, in trained rats the collagen accumulation in interstitial tissue of heart was 3.2 ± 0.1% after stanozolol, 3.1 ± 0.1% after cannabis, and 4.3 ± 0.4% after both substances administration in comparison to 0.13 ± 0.03% in control trained rats (*p* < 0.05, Fig. 3E–H).

### Effect of Stanozolol and Cannabis Misuse on Cardiac Tissue Oxidative Stress Biomarkers

**Fig. 4:**
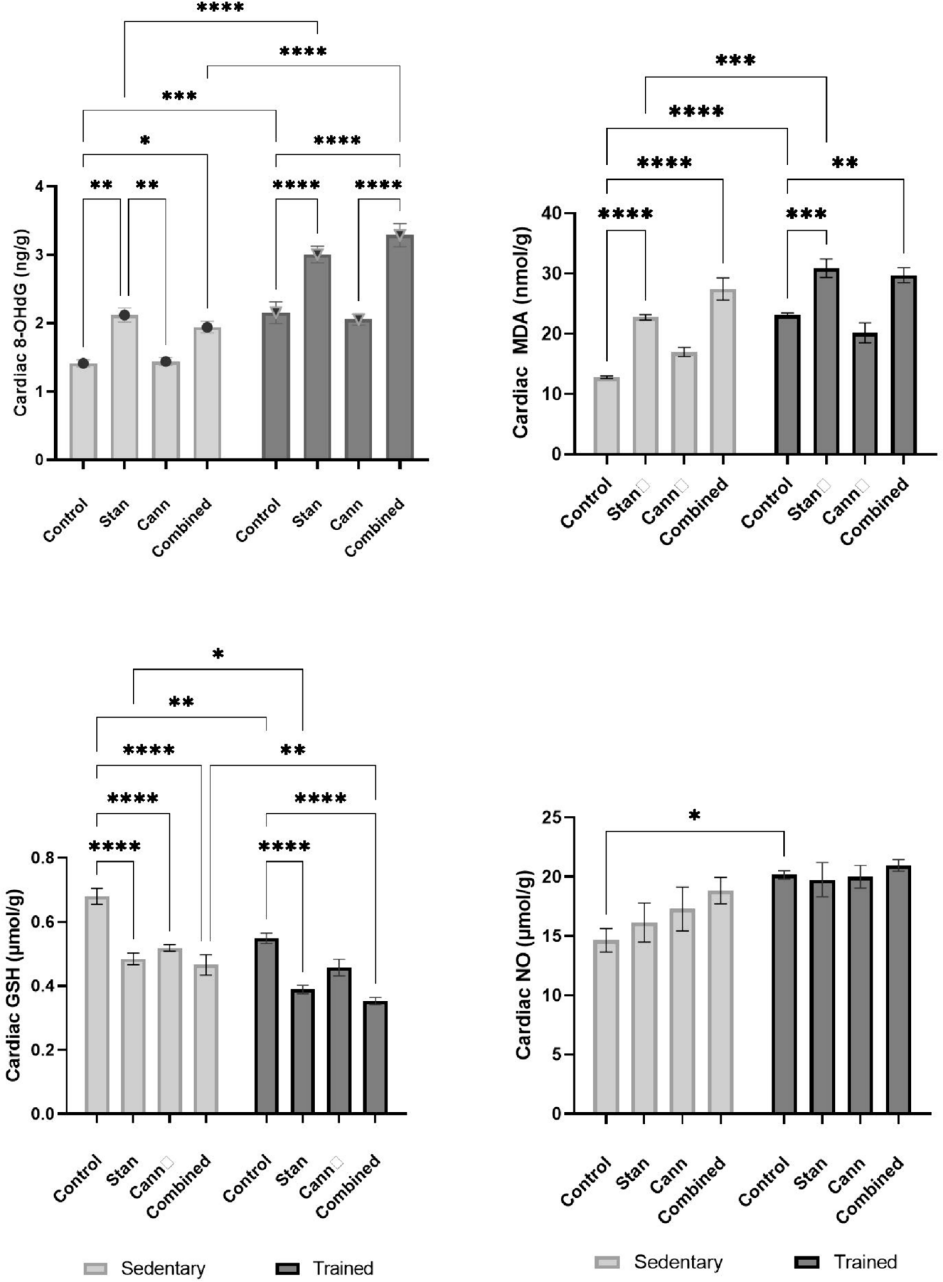
Effects of stanozolol administration and cannabis administration on cardiac oxidative biomarkers in sedentary or trained rats. **A** 8-OHdG, **B** MDA, **C** GSH D) NO. Rats were administered stanozolol 5 mg/kg, s.c. (Stan), or cannabis resin extract 10 mg/kg, i.p. (Cann), or both substances (Combined) for a period of 8 weeks. A set of groups was kept sedentary and the other set was trained by swimming protocol. The results are expressed as mean ± SEM; Significance was tested at *p* < 0.05 using two-way ANOVA followed by Tukey's post hoc test for comparison (*n* = 6/group). *, **, ***, **** compared to the corresponding set control at *p* < 0.05, 0.01, 0.001, 0.0001, respectively

Two-way ANOVA of cardiac 8-OHdG content showed a factor interaction accounting for 4.21% of the total variance of 8-OHdG *F*(3, 40) = 4.10, *p* = 0.013, while the training accounted for 44.51% of the total variance *F*(1, 40) = 130.00, *p* < 0.0001 and the treatments accounted for 37.58% of the total variance *F*(3, 40) = 36.59, *p* < 0.0001. A significant increase of its level in heart tissue of rats administered stanozolol alone or in combination with cannabis was observed in either sedentary (Stan: 2.12 ± 0.1 ng/g tissue, mean difference: − 0.71, CI − 1.21 to − 0.20, *p* < 0.01, combined: 1.91 ± 0.08 ng/g tissue, mean difference: − 0.53, CI − 1.03 to − 0.03, *p* < 0.05,) or trained rats (Stan:3.01 ± 0.12, mean difference: − 0.85, CI − 1.35 to − 0.35, *p* < 0.0001, combined: 3.01 ± 0.02 ng/g tissue, mean difference: − 1.13, CI − 1.64 to − 0.63, *p* < 0.001) compared to the control group (sedentary 1.14 ± 0.05, trained: 2.15 ± 0.15 ng/g tissue). Cardiac content of 8-OHDG was not affected by cannabis administration (sedentary: 1.42 ± 0.05 ng/g tissue, mean difference: 0.03, CI − 0.53 to 0.47, *p* > 0.05, trained: 2.05 ± 0.08 ng/g tissue, mean difference:0.10, CI − 0.40 to 0.60, *p* > 0.05). Figure 4.

Two-way ANOVA of data of lipid peroxidation product, MDA showed interaction between the two factors, training and treatments *F*(3, 40) = 5.45 (*p* = 0.003) exhibiting a clear effect of the swimming training *F*(1, 40) = 51.96 (*p* < 0.0001) and different treatments across the groups *F*(3, 40) = 44.09 (*p* < 0.0001), Fig. 4. In sedentary rats, stanozolol administration increased the cardiac tissue level of MDA compared to control rats (mean difference: − 9.94, CI − 15.24 to − 4.63, *p* < 0.001), and the administration of both stanozolol and cannabis (mean difference: 14. 65, CI − 19.95 to − 9.35, *p* < 0.0001, Fig. 4). Swimming training induced the MDA content compared to sedentary rats (mean difference: 10.31, CI 5.01–15.61, *p* < 0.0001, Fig. 4). Stanozolol administration of trained rats resulted in a significant increment (mean difference: − 7.79, CI − 13.0 to − 2.5, *p* < 0.001) and likewise the combined treatments (mean difference: − 6.64, CI − 11.94 to − 1.34, *p* < 0.01, Fig. 4). Noteworthy, the MDA level in heart of trained rats receiving stanozolol was significantly higher than its corresponding concentration in sedentary animals (mean difference = 8.14, CI 2.841–13.44, *p* < 0.001), while there was no significant difference between sedentary and trained rats receiving combined treatment (mean difference: 2.29, CI − 3.0 to 7.59, *p* > 0.05). Cannabis administration showed no significant difference of MDA levels in sedentary and trained rats (mean difference of sedentary groups: − 4.20, CI − 9.49 to 1.09, mean difference of trained groups: 2.95, CI − 2.34 to 8.25, *p* > 0.05, Fig. 4).

In addition, two-way ANOVA of the antioxidant GSH content showed no interaction of training and treatment (1.5%, *p* > 0.05). However, training was of significant effect on cardiac GSH content (22.9%, *p* < 0.0001). The antioxidant GSH repository concentration was significantly affected by training (mean difference = − 0.13, CI − 0.22 to − 0.04, *p* < 0.01). Different Treatments accounted for 56.5% of the total variance, stanozolol was found to reduce its content in sedentary rats (mean difference: 0.19, CI 0.10–0.29, *p* < 0.0001) and the heart tissue of trained rats (mean difference: 0.16, CI 0.07–0.25, *p* < 0.0001). Sedentary cannabis group showed a significant inhibition of cardiac GSH content compared to sedentary control group (mean difference: 0.16, CI 0.07–0.25, *p* < 0.0001), while it was not significant in trained rats (mean difference: 0.09, CI − 0.0007 to 0.18, *p* > 0.05). Combination of both substances resulted in a comparable decrement of GSH (mean difference against sedentary control = 0.21, 95% CI 0.12–0.31, *p* < 0.0001, mean difference against trained control = 0.19, 95% CI 0.10–0.28, *p* < 0.0001 (Fig. 4).

On the other hand, two-way ANOVA of cardiac NO content revealed no interaction between training and treatment (3.5%, *F*(3, 40) = 0.75, *p* = 0.5), a significant training effect *F*(1, 40) = 16.64, *p* = 0.0002, and no significant treatment effect *F*(3, 40) = 1.59, *p* = 0.2070. There was no significant change in cardiac NO content after different treatments in sedentary and trained rats (*p* > 0.05), Fig. 4, but training increased the NO content in control rats (mean difference = 5.52, 95% CI 0.05–10.99, *p* < 0.001). Figure 4.

## Discussion

Hormonal disturbances resulting from abuse of AASs is well-studied, however cardiovascular toxic effects need further studies [24], particularly the practice of young and adult sport trainers of misusing what is called image- and performance-enhancing drugs with taking multiple drugs in unsupported dosing regimens making this practice obscure [25]. Among these performance-enhancing drugs, stanozolol and cannabis were the drugs of choice in the current study based on recent data about the most misused drugs in sports [26]. Additionally, there were many reported death cases linked to stanozolol abuse [27–29] encouraging full health awareness about this practice.

The current study demonstrated a deleterious hypertrophic effect on cardiac tissue of male wistar rats after cannabis and or stanozolol administration with or without exercise. In this study, an increase in relative heart weight (RHW) was observed in response to stanozolol and cannabis. Our findings concur with the report of Barbosa dos Santos [19] who similarly noted higher RHW after exposing rats to stanozolol and exercise. Overuse of AAS has been linked to hypertrophy and alterations in cardiomyocytes that can occasionally be irreversible, such as concentric left ventricular hypertrophy [30] Primarily, AAS facilitate the build-up of muscles through up-regulating the androgenic mediated gene transcription [31]. The expression of androgenic receptors can be augmented by resistance exercise performance as well as prolonged exposure to AAS [32]. This process could be a potential mechanism by which exercise and large doses of AAS could cause heart hypertrophy [33–37].

The cellular pathology and organ physiology alterations that follow stanozolol or cannabis treatment with or without exercise are comparable to those seen in cardiomyopathy and heart failure. Supra physiological doses of AAS could lead to pathological heart hypertrophy and histopathological changes that has been linked to ventricular remodeling and sudden cardiac mortality [38, 39]. Recently, Mohammed Hassan et al. and Hanan et al. observed hypertrophy and degeneration in the skeletal and cardiac muscles after nandrolone decanoate [40, 41]. Moreover, Smit et al. [42] described a broadening of the interstitial tissue in the cardiac muscles of rats treated with AAS. Previous data showed that AAS caused left ventricular hypertrophy in recreational strength athletes, preceding compromised systolic and diastolic function [43]. Chronic low-dose or acute high-dose stanozolol treatment resulted in left ventricular hypertrophy [44], and another study reported that chronic administration of testosterone significantly increased RHW of rats [37].

Collagen fiber content is another crucial component in heart hypertrophy. The heart’s collagen content may be considerably raised by the combination of stanozolol and exercise, where both promote the production of collagen in cardiac muscles [45]. Additionally, both stanozolol and cannabis with or without training stimulated COL3 accumulation in cardiac muscle, which is a structural protein of the cardiac collagen matrix [46]. Cardiac fibroblasts are described as the primary source of myocardial COL1 and COL3 peptides [47, 48]. High circulating COL3 is correlated with left ventricular hypertrophy [49], where the induced connective tissue collagen fiber denotes cardiac hypertrophy and decreased compliance of the ventricular wall that can eventually result in heart failure [50].

Our findings demonstrated that use of stanozolol and cannabis induced VEGF in heart tissue and the histological observations showed microvasculature presence in the hypertrophic left ventricle. Excessive neovascularization with hyper-permeable vessels could directly contribute to fibrotic changes observed the present investigation. Cardiomyocytes are known to produce the endothelial cell mitogen (VEGF-A) to regulate microvascular permeability and promote cardiac angiogenesis. Nonetheless, it acts directly on the endothelium and promotes extravasation of plasma fibrinogen, leading to fibrin deposition that alters the tissue extracellular matrix. This is particularly observed after ischemic insults [42]. and Zhao et al. and Heba et al. [50, 51] reported that VEGF mRNA was highly expressed in the infarcted myocardium and lasted for 6 weeks.

Androgenic receptors are known to modulate cardiac remodeling and fibrosis associated with a hypertrophic stress [52]. Generally, androgens produce metabolic effects through different processes mediated by G protein coupled receptors affecting downstream pathways such as CaMKII, ERK1/2, and AMP-activated protein kinase (AMPK) [53].G1 coupled activation suppress the TGF-β1/SMAD2 fibrotic gene transcription and reduce the production of collagen at physiological androgen doses while increasing the risk of apoptosis at supra physiological levels. The inhibition of Erk, Akt, and CaMKII trigger transcription of hypertrophy related genes in cardiac muscle. Erk1/2 signaling is important in the process of extracellular matrix proteins accumulation such as collagen deposition[54]. Furthermore, cannabinoid receptors are G-protein coupled receptors that influence the connected other pathways MAPK, ERK. The cannabis active components caused an upregulation in phosphorylation of AKT and ERK1/2 through G-protein coupled receptor activation [55]. In addition, oxidative stress is exaggerated in such conditions. This pathway may have played a role in the findings of the present study since it could have been affected by both stanozolol and cannabis synergistically.

A common consequence of these fibrotic changes is the tissue inability to maintain a healthy redox balance and induced activity of free radicals observed as enhanced lipid peroxidation, reduced GSH antioxidant defense and consequently, protein degradation and DNA fragmentation, expressed as 8-OHdG content.

Indeed, it is well known that overproduction of free radicals and lipid peroxidation are linked to the development of different forms of cardiac injury since they contribute to endothelial dysfunction [56]. Reactive oxygen species (ROS) production during exercise in response to oxidative stress is another method of evaluating myocardial damage [57]. Previous research showed that a relatively anoxic state similar to an ischemia–reperfusion state occurred after every exercise set in the exercising muscle in one explanation. Other potential mechanisms were suggested including xanthine–xanthine oxidase pathway, lactic acid-induced production of hydroxyl radical, neutrophil respiratory burst, catecholamine autooxidation, and altered calcium homeostasis that could be accountable for the exercise-induced ROS formation [58].

8-OHdG was examined as a biomarker of oxidative DNA damage in connection to changes in oxidative stress indicators [32]. The finding of the present work are in parallel with a previous report which showed obvious tissue inflammation, cardiac muscle fiber necrosis, and hyperplasia [57]. Our previous data described redox imbalance in heart tissue of rats receiving supraphysiological doses of anabolic steroids [59].

In this study, 8-OHdG excretion significantly increased after 8 weeks of training with stanozolol and cannabis dosing regimen. Training increased the level of 8-OHdG in previous studies [60, 61], while in another study it was not altered after squat exercise. Conversely, several research findings suggested that neither submaximal nor maximum exercise was associated with an increase in DNA damage [62]. Although this discrepancy is still debated, it can be explained in the light of the length and intensity of exercise, athletic fitness level, or other factors which can affect the severity of oxidative stress.

Measuring the MDA levels in cardiac homogenate serves as a useful biomarker of tissue oxidative stress and provides a convenient index of lipid peroxidation. In our study, stanozolol and cannabis administration to sedentary or exercising rats for an extended period of time (8 weeks) caused a significantly higher cardiac level of MDA and a decrease in the cardiac content of GSH. Cannabis was proven to be related to lipid peroxidation, where long-term cannabis smokers exhibited elevated blood MDA level, and diminished GSH level [63].

And urinary 8-OHdG compared to healthy controls. Other experimental studies showed disturbed oxidative biomarkers in rats given a two-week marijuana extract treatment [64–66].

Exposure to exercise induced MDA in the current study. It was shown that a significant increase in plasma MDA concentration followed a circuit type exercise [67]. It has been reported that because of polyunsaturated fatty acids content in cellular membranes, the exercise-induced muscle damage cause a leak of polyunsaturated fatty acids in the blood thus produce significant elevation of the oxidation product, MDA [68].

In this study there were no changes in cardiac level of (NO) in all groups. Our finding was in agreement with the results of Rocha [44]. On the other hand, previous studies detected altered NO metabolism following different training exercises [69]. Testosterone derivatives stimulated NO production by up-regulating the expression of eNOS and ERK1/2 signaling [70].

## Conclusion

The presented study noted that eight weeks of treatment with stanozolol and/or cannabis, with or without physical training, up regulated the oxidative stress damage biomarkers, induced cardiac hypertrophy, elevated fibrotic markers, and resulted in higher cardiac proliferation in wistar rats. Our findings provide a fresh viewpoint on the cardiac harm caused by stanozolol and/or cannabis. In other words, oxidative stress and a multitude of heart-related risk factors should be taken into account as underlying causes. Nevertheless, each of these elements had a part in the emergence of the harmful effects of stanozolol and/or cannabis on cardiac tissue, and they will continue to be a source of interest for long-term drug addiction heart study. To confirm the connection between elevated oxidative stress damage indicators and AAS-induced sudden cardiac mortality, more research and clinical retrospective follow-up studies are required. In addition, it is a first-time report of the added effect of cannabis co-abuse with stanozolol use though commonly abused emphasizing the current risks faced by either professional or amateur sport trainers. Since limited clinical data are present and ethical considerations forbid conducting such toxic and life-threatening studies, it is necessary to increase awareness about the cardiovascular burden of such practices among these communities.

## Data Availability

Data will be made available on request.
